# An integrated strategy combining metabolomics and machine learning for the evaluation of bioactive markers that differentiate various bile

**DOI:** 10.3389/fchem.2022.1005843

**Published:** 2022-10-19

**Authors:** Xinyue Li, ChenRui Liang, Rui Su, Xiang Wang, Yaqi Yao, Haoran Ding, Guanru Zhou, Zhanglong Luo, Han Zhang, Yubo Li

**Affiliations:** ^1^ State Key Laboratory of Component-Based Chinese Medicine, Tianjin University of Traditional Chinese Medicine, Tianjin, China; ^2^ Key Laboratory of Pharmacology of Traditional Chinese Medicine Formulae, Ministry of Education, Tianjin University of Traditional Chinese Medicine, Tianjin, China; ^3^ School of Chinese Materia Medica, Tianjin University of Traditional Chinese Medicine, Tianjin, China; ^4^ Pharmacy Faculty, Hubei University of Chinese Medicine, Wuhan, Hubei, China; ^5^ College of Chemistry and Molecular Sciences, Wuhan University, Wuhan, Hubei, China

**Keywords:** bile acids, key markers, machine learning, anti-inflammatory activity, metabolomics

## Abstract

Animal bile is an important component of natural medicine and is widely used in clinical treatment. However, it is easy to cause mixed applications during processing, resulting in uneven quality, which seriously affects and harms the interests and health of consumers. Bile acids are the major bioactive constituents of bile and contain a variety of isomeric constituents. Although the components are structurally similar, they exhibit different pharmacological activities. Identifying the characteristics of each animal bile is particularly important for processing and reuse. It is necessary to establish an accurate analysis method to distinguish different types of animal bile. We evaluated the biological activity of key feature markers from various animal bile samples. In this study, a strategy combining metabolomics and machine learning was used to compare the bile of three different animals, and four key markers were screened. Quantitative analysis of the key markers showed that the levels of Glycochenodeoxycholic acid (GCDCA) and Taurodeoxycholic acid (TDCA) were highest in pig bile; Glycocholic acid (GCA) and Cholic acid (CA) were the most abundant in bovine and sheep bile, respectively. In addition, four key feature markers significantly inhibited the production of NO in LPS-stimulated RAW264.7 macrophage cells. These findings will contribute to the targeted development of bile in various animals and provide a basis for its rational application.

## Graphical Abstract

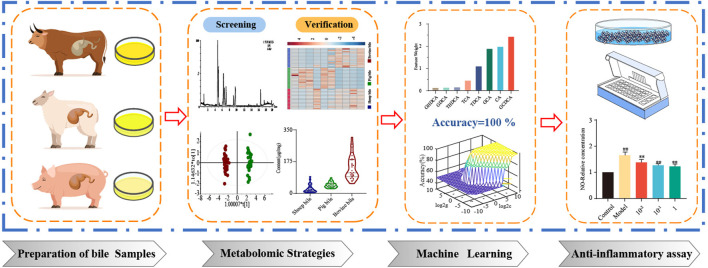

## 1 Introduction

As a kind of natural medicine, animal medicine is an important part of traditional Chinese medicine because of its abundant resources and curative effects (Feng et al., 2009). Animal bile has been used as a medicine for thousands of years. Nearly 30 kinds of animal bile are contained in the “Compendium of Materia Medica”, including birds, reptiles, mammals, etc. It is bitter in taste and cold-natured, with the effects of eyesight, antispasmodic, clearing heat, and cooling, and detoxification (Qiao et al., 2011). Pig bile, bovine bile, and sheep bile are the most commonly used and readily available bile medicines. However, there are great differences in the pharmacological effects and main applications of various types of bile. The misuse of bile by some producers results in the uneven quality of animal bile herbs and disorder in the market. This phenomenon seriously affects economic interests and endangers the health of consumers (Xiong et al., 2019). Most of the available literature reports focused on the determination of one animal bile with a lack of studies on the simultaneous determination of components in multiple animal bile. Therefore, there is an urgent need to establish accurate analytical techniques for the isolation and identification of these species. It is of great significance for its processing and utilization as well as for ensuring its quality and curative effect.

Bile acids are the most abundant bioactive substances in bile, which are synthesised from cholesterol in hepatocytes by the catabolic pathway, comprising primary and secondary bile acids (Cao et al., 2022). Its structure contains a steroid nucleus with a valeric acid side chain attached to the C-17 position (McGlone and Bloom 2019). In recent years, numerous studies have found a significant medicinal value for bile acids, such as regulating lipid metabolism and glucose metabolism and treating cholestatic liver disease, obesity, type 2 diabetes, dyslipidemia, and nonalcoholic steatohepatitis (Li and Chiang 2014; Arab et al., 2017; Chavez-Talavera et al., 2017). Currently, Ursodeoxycholic acid (UDCA) and Chenodeoxycholic acid (CDCA) are widely used as listed medicines (Fiorucci and Distrutti 2019). With the widespread application of bile acids in the pharmaceutical industry, the demand for animal bile has increased. The preparation of bile acids is mainly obtained by natural bile extraction (Chanquia et al., 2018; Namegawa et al., 2018). However, bile from different animals is difficult to distinguish, and the major bile acids present in each bile as well as the biological activities of bile acids are not yet clear, which greatly limits the processing and reuse of bile. Therefore, it seems to be crucial to distinguish animal bile to improve the sustainable development of the industrial chain. The screened chemical markers can provide a basis for the targeted development of the bile acids of bile from different animals.

Recently, metabolomics has been widely used in the field of traditional Chinese medicine, including quality evaluation of medicinal materials, chemical composition analysis, toxicity mechanism research, and pharmacodynamic material mechanism research (Schrimpe-Rutledge et al., 2016; Chu et al., 2020; Ma et al., 2020; Zhang et al., 2020). In the pharmaceutical field, it is a tool for finding differential markers and distinguishing different types of pharmaceutical products. It includes a variety of chemometric methods, such as orthogonal partial least-squares discrimination analysis (OPLS-DA) and hierarchical clustering analysis (HCA), which have been widely used in various analytical research fields (Liu et al., 2019; Li et al., 2020). This also provides the basis for the screening of differential markers. However, the volume of data collected by metabolomics is large, and the similarity of most samples is high, which makes it difficult to achieve consistency evaluation and identification of unknown samples only by omics approaches (Liang et al.,^2022^). As a result, smarter and more efficient means of processing data are needed. In this case, machine learning is a good choice, which can largely compensate for the lack of data classification and processing (Woolley et al., 2021).

In this study, a multivariate statistical method based on UPLC-Q-TOF/MS was used to screen the chemical markers of bile from different animals. Second, neighborhood component analysis (NCA) was adopted to extract the most effective key feature markers, and a support vector machine (SVM) algorithm was combined to verify the accuracy and specificity of the key markers. Finally, the contents of four key feature markers were accurately quantified, and their anti-inflammatory activities were evaluated. This is the first time that the “metabolomic strategy-machine learning-anti-inflammatory cell assay” has been combined for an in-depth and comprehensive comparison and analysis of bile acids in bile from various animals. This study provided a useful reference for the development and utilization of key markers in the bile of different animals.

## 2 Materials and methods

### 2.1 Chemicals and reagents

30, 32, and 31 batches of fresh pig bile, sheep bile, and bovine bile were purchased from farmers’ markets in Tianjin. HPLC-grade methanol was obtained from Tianjin Concord Technology Co., Ltd. (Tianjin, China). HPLC-grade formic acid was acquired from Thermo Fisher (Shanghai, China). GCA, TDCA, and GCDCA were purchased from Shanghai Macklin Biochemical Co., Ltd. (Shanghai, China); CA and taurochenodeoxycholic acid (TCDCA) were obtained from Dalian Meilun Biotechnology Co., Ltd. (Dalian, China); CDCA was procured from Meryer Chemical Technology Co., Ltd. (Shanghai, China); glycodeoxycholic acid (GDCA), glycohyodeoxycholic acid (GHDCA), and sodium taurocholate (TCA-Na) were purchased from Shanghai Yuanye Bio-Technology Co., Ltd. (Shanghai, China); tauro hyodeoxycholic acid (THDCA) was obtained from Sollerbauer Technology Co., Ltd. (Beijing, China).

### 2.2 Screening for chemical markers among the bile of different animals

#### 2.2.1 Sample preparation

The bile was filtered through gauze and then prefrozen at **−**80°C for 12 h. All samples were lyophilized in a vacuum freeze-dryer (EYELA, TOKYO RIKAKIKAI CO., LT D, FDU-2110) for 48 h. Approximately 5 mg of bile powder was weighed and extracted by ultrasonication for 45 min with a mixed solvent (water containing 1% formic acid mixed with methanol in a volume ratio of 5:95). The solution was fixed in a 10 mL volumetric flask, mixed well, and filtered through a microporous membrane (0.22 μM) before use (Li et al., 2022). All bile samples were mixed to obtain a quality control sample (QC).

#### 2.2.2 Chromatography and mass spectrometry

The separation was performed on an ultrahigh-performance liquid chromatography (UPLC) system (I-Class, Waters Corp., Manchester, United Kingdom) combined with a quadrupole time-of-flight mass spectrometer (Q-TOF/MS, Xevo G2-S, Waters Corp., Manchester, United Kingdom). The mobile phase consisted of a 0.1% formic acid aqueous solution (A) and a 0.1% formic acid acetonitrile solution (B). The gradient elution procedure used was as follows: 0–0.5 min, 5% B; 0.5–1 min, 5% B→20% B; 1–3 min, 20% B→40% B; 3–12 min, 40% B→60% B; 12–16 min, 60% B→90% B; 16–17 min, 90% B→100% B; 17–18 min, 100% B; 18–19 min, 100% B→5% B; and 19–20 min, 5% B. The flow rate was 0.25 mL/min, and the injection volume was 5 μL. The column and autosampler were maintained at 40°C. An electrospray ion source (ESI) was used to perform a full scan in the negative ion mode. Data collection ranged from 50 to 1000 Da. The operating parameters of the ESI source were as follows: capillary voltage, 2.0 kV; cone voltage, 30 V; cone gas flow rate, 50 L/h; ion source temperature, 100°C; and dissolvent gas temperature, 450°C.

#### 2.2.3 Method validation for metabolomics study

1) Instrumental precision: The same QC sample was injected for 6 times, 10 peaks were randomly selected and the RSD values of peak area and retention time were calculated for each of the 10 peaks.
2) Method precision: Six QC samples were prepared in parallel and injected for analysis. 10 peaks were randomly selected and the RSD values of peak areas and retention times of the 10 peaks were calculated.
3) Sample stability test: The same QC sample solution was sampled at 0, 6, 12, and 18 h. Ten peaks were randomly selected and the RSD of peak area and retention time were calculated for each of the ten peaks.

#### 2.2.4 Multivariate statistical analysis of bile from different animals

To compare and distinguish the bile of each species, UPLC-Q-TOF/MS was used for sample analysis in this study. First, raw data were exported to MassLynx version 4.1 software (Waters Corp., Milford, MA), and the data provided the m/z value and retention time as well as the detected peak with a certain intensity. Then, the data were normalized and imported into SIMCA-P14.1 (Umetrics AB, Sweden) for multivariate data analysis. The aggregation degree of bile can be determined from the score plot of principal component analysis (PCA). To clarify the differences between them, chemical markers of the bile from three animals were screened. Then, an OPLS-DA model of the samples was performed, and the variable importance projection (VIP) of each chemical component was calculated. A variable whose VIP was greater than 1.0 was usually considered to be a potential marker (Li et al., 2020). Therefore, the components with VIP>1 were screened and subjected to statistical analysis by SPSS 20.0, and chemical markers with statistically significant differences were obtained (*p* < 0.05).

### 2.3 Establishment of machine learning models

To further validate the accuracy of the chemical markers, NCA analysis of the chemical markers was performed using MATLAB (MathWorks, United States). The input variables were as follows: sheep bile labeled “1”, bovine bile labeled “2”, and pig bile labeled “3”. The SVM machine learning models were built with the marker values as input variables and the response values of key markers as feature variables. Samples were randomly divided into a training set (2/3) and a test set (1/3) for model building and accuracy verification. Thus, the accuracy of key markers was further validated.

### 2.4 Quantitative analysis of key markers

To further determine the distribution of screened key markers in each bile, UPLC (1290 Infinity, Agilent Technologies) combined with QQQ-MS (QTRAP ®6500, AB SCIEX) was used for quantitative analysis in this study. Chromatographic separation was achieved on a UPLC BEH C18 column (Waters ACQUITY, 100 mm × 2.1 mm × 1.7 μm). The mobile phase consisted of a 0.01% formic acid aqueous solution (A) and a 0.01% formic acid acetonitrile solution (B). The elution program was as follows: 0–2 min, 10% B; 2–3.5 min, 10% B→50% B; 3.5–9 min, 50% B→70% B; 9–10.5 min, 70% B→90% B; 10.5–12 min, 90% B; 12–12.1 min, 90% B→10% B; 12.1–15 min, 10% B. The total analysis time was 15 min, the injection volume was 2 μL, and the flow was retained at 0.3 mL/min. The ESI source worked in negative ion mode under the following conditions. Curtain gas (CUR) 30, nebulizing gas (GS1) 55, drying gas (GS2) 55, ion spray voltage (IS) −4.5 kV, ion source temperature (TEM) 550°C, declustering potential (DP)-130.0, entrance potential (EP)-10.0, collision cell exit potential (CXP)-13.0. Data were analyzed by Analyst 1.6.3 (AB SCIEX, United States).

### 2.5 Anti-inflammatory activity research of key markers

#### 2.5.1 Dual-luciferase reporter gene system of 293T cells for the detection of NF-κB expression

Before the luciferase experiments, cell viability under different concentrations of key markers was first investigated. 293T cells at the logarithmic growth stage were inoculated in 96-well cell culture plates at a density of 2 × 10^5^ cells/mL. Then, 100 μL of 0.01, 0.1, 1, 10, and 100 μM CA, GCA, GCDCA, and TDCA were added to each well in 4 parallels. After 24 h of treatment, the supernatant was aspirated, and CCK-8 solution was added to each well and then incubated for 30 min at 37°C. The absorbance OD value was measured at 450 nm by an enzyme marker.

The NF-κB luciferase reporter plasmid (pGL4.32) and PGMLR-TK Renilla Fluorescein Reporter Plasmid (pGL4.75) were simultaneously transfected into 293T cells with PEI (1 mg/mL) transfection reagent. Cells transferred into plasmid were cultured for 24 h, and then dexamethasone (10 μM) and the key marker with a safe concentration were separately added. Control and Model groups were set up and incubated for another 6 h. The cells were then lysed and assayed with the Dual-Luciferase assay system. Six parallel wells were set up and replicated three times. Finally, the relative luciferase activity was obtained by comparing firefly luciferase activity with Renilla reniformis luciferase activity.

#### 2.5.2 Effect of key markers on NO production in LPS-stimulated RAW264.7 macrophages

First, cell viability assays were performed on RAW264.7 cells. RAW264.7 cells at the logarithmic growth stage were collected and added to 0.01, 0.1, 1, 10, 100 μM CA, GCA, GCDCA, and TDCA solutions. Four parallel wells were set for each concentration, and the control group and DMSO group without drugs were set at the same time. After 24 h of dose addition, 100 μL CCK-8 solution was added to each well and incubated at 37°C for 30 min. The absorbance OD value was measured at 450 nm with a microplate reader, and the cell viability was calculated.

RAW264.7 cells were treated with LPS at a concentration of 1 μg/mL and CA, GCA, GCDCA, and TDCA at a final concentration of 0.1 μM. After 24 h of dose addition, the medium supernatant was collected from microporous plates. Then, the solution was mixed with the same amount of Griess reagent, and the absorbance value of the solution was measured at 540 nm. The concentration of NO in the samples was calculated according to the standard curve.

## 3 Results and discussion

### 3.1 Identification and comparison of the bile from various animals

**FIGURE 1:**
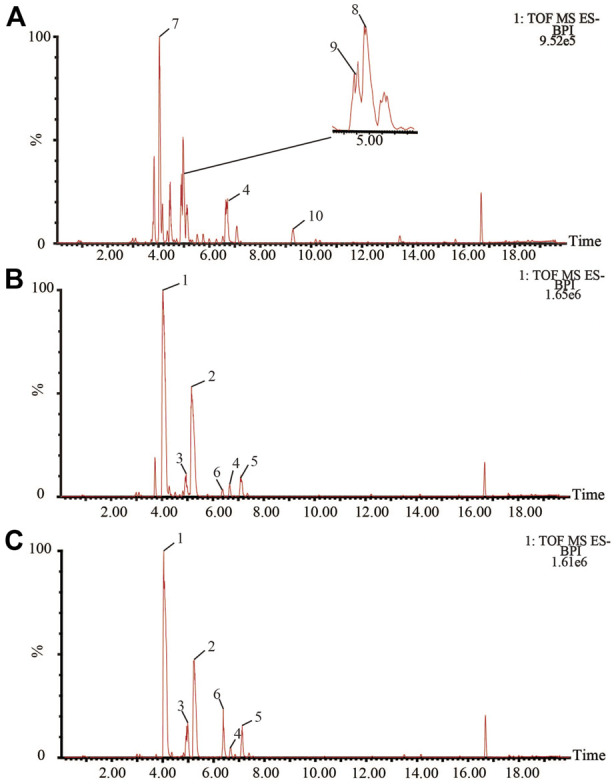
BPI chromatograms of bile **(A)**: pig bile; **(B)** bovine bile; **(C)** sheep bile in negative ESI mode. 1 TCA; 2 TDCA; 3 GCA; 4 GCDCA; 5 GDCA; 6 CA; 7 THDCA; 8 TCDCA; 9 GHDCA; 10 CDCA.

Bile acids are the main active components of bile. UPLC-Q-TOF/MS was used to collect the spectral information of each bile sample in negative ion mode, and the BPI chromatograms of QC were shown in Figure 1. The RSD values of the chromatographic peak areas were less than 15% and the RSD values of the retention times were less than 3% (Supplementary Table S2–S7). The results showed that the instrument precision, and the method precision were good, the samples were stable during the injection process. As many bile acid components are isomers of each other, reference substances are required for comparison. The sample profiles were compared with the profiles of dozens of bile acid reference substances available in the laboratory and 10 bile acids were accurately identified (Supplementary Table S1). Where TCA, TDCA, GCA, GDCA, GCDCA, GHDCA, THDCA, and TCDCA summed in a [M-H]^-^ manner and CA, CDCA summed in a [2M-H]^-^ manner.

**FIGURE 2:**
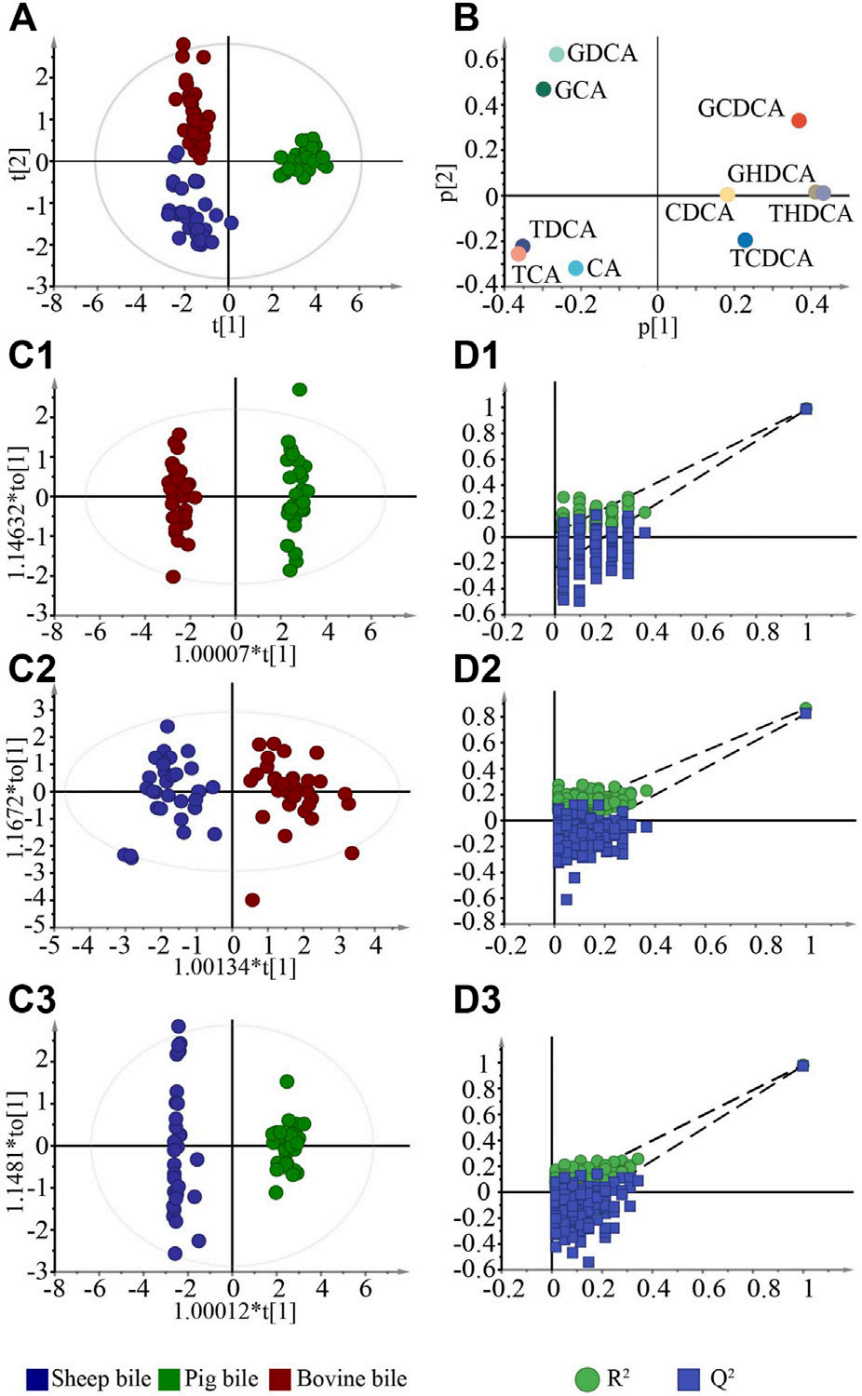
Multivariate statistical analysis for bile in negative ion mode. **(A)**. PCA score plots; **(B)**. Loading plots of components; **(C1–C3)**. OPLS-DA score plots between various bile acids; **(D1–D3)**. Permutation test of the OPLS-DA models.

To visualize and compare the differences in bile between different animals, the PCA model was established (Figure 2A). The PCA score plot results showed a clear trend toward separation among pig bile, bovine bile, and sheep bile. The contribution of each marker to distinguishing between different bile was then further analyzed by loading plot (Figure 2B). The greater the absolute value of each component in the coordinate system, the greater the contribution to differentiating the bile of different animals.

### 3.2 Screening of chemical markers among various bile

OPLS-DA is a supervised classification technique that filters out discrepant components from a dataset to further enhance its interpretation (Guo et al., 2018). OPLS-DA models were established to screen the chemical markers that distinguish between pig, bovine, and sheep bile (Figures 2C1–C3). The models could enhance the separation trend by reducing extraneous noise in the data. The R^2^Y of the models ranged from 0.863–0.990, and the Q^2^ ranged from 0.823–0.985. The majority of the samples fell within the 95% confidence interval, indicating that the models were stable and reliable. Then, the models were subjected to a permutation test 200 times. As shown in Figures 2D1–D3, all models had good fitting and prediction ability. After statistical analysis, components with VIP >1 and a significant difference in the content (*p* < 0.05) were considered chemical markers. There were six chemical markers between bovine bile and pig bile, which were THDCA, GDCA, TCA, TDCA, GCA, and GHDCA. Five chemical markers were screened between bovine bile and sheep bile (GCDCA, GDCA, TCA, GCA, and CA), and six chemical markers were screened between sheep bile and pig bile (TCA, THDCA, TDCA, GHDCA, GCDCA, and GCA).

The OPLS-DA results showed a large difference in the composition of the three bile acids, which could be easily distinguished. These differences may be caused by different animal species, habits, and living environments. Thus, these chemical markers are the material basis for the differences in bile quality between animals.

### 3.3 Screening and validation of key feature markers

#### 3.3.1 Screening of key feature markers

According to the OPLS-DA models, we initially screened eight bile acids that could distinguish three kinds of bile. However, when establishing a machine learning model, not every feature contributes significantly to the predicted target value (Kaplan et al., 2021). Removing low contributing variables and using key feature markers to construct machine learning models can improve the generalization ability of the models. NCA is an effective means of finding key features and is commonly used by researchers for feature selection when modeling against high-dimensional data (Jiménez-Grande et al., 2021). In this study, NCA was used to select key features for chemical markers between the bile of different animals, and key feature markers were modeled as feature variables. The weight values for each feature are shown in Supplementary Figure S1. The features with higher weights were the ones that were more significant to the classification value. The weights of TDCA, GCA, CA, and GCDCA were all greater than 1, indicating that these four components contributed more to differentiating the bile of different animals. All four components have been shown to have antibacterial, anti-inflammatory, antihypertensive, anti-cough, and expectorant pharmacological effects. Previous reports showed that these four feature markers had pharmacological effects, such as bacteriostasis, anti-inflammatory, antihypertensive, antitussive, and expectorant effects (Zhou et al., 2021).

#### 3.3.2 Validation of key feature markers

To validate the accuracy and specificity of the key feature markers, a polynomial kernel function classifier (PL-SVM) model was established using these four feature markers as feature variables. The classification performance of SVM is affected by many factors, among which the following two factors are crucial: 1) the error penalty Factor C and 2) the form of the kernel function and its parameters (Maltarollo et al., 2019). The polynomial kernel function that we chose for modeling is one of the most widely used and applicable kernel functions (Ye et al., 2013). In this study, the grid search method was used to perform a global search for optimization of the parameters C and g to achieve the best discriminative performance of the PL-SVM model. When C was 0.134 and g was 3.732, the PL-SVM model had the most accurate discriminant performance.

In addition to g, the main parameters of the PL-SVM model also include the polynomial coefficient and coef 0. The optimal g was determined by the grid search method, and then an optimization search was performed for the polynomial coefficients as well as the coef 0 value. The results showed that the PL-SVM models with optimal performance were obtained when the polynomial coefficients were 3 and 4, the models were fitted with 100% CV accuracy, and the classification accuracy was up to 100% (Supplementary Table S8). However, the larger the polynomial coefficients are, the higher the dimensionality of the mapping and the consequent increase in computational effort. When the coefficients are too large, learning is too complex and tends to lead to overfitting (Ellmann et al., 2020). The factor of 3 was chosen to take into account the size of the calculation and the amount of memory used. The model performed best when the polynomial coefficient was 3 and coef 0 was 7.4, with a CV accuracy of 100% and a classification accuracy of 100%. The results showed that the established PL-SVM model was stable and reliable and could accurately differentiate bile from different sources. In addition, the four key feature markers were highly accurate and specific.

### 3.4 Distribution of key feature markers in the bile of three animals

**FIGURE 3:**
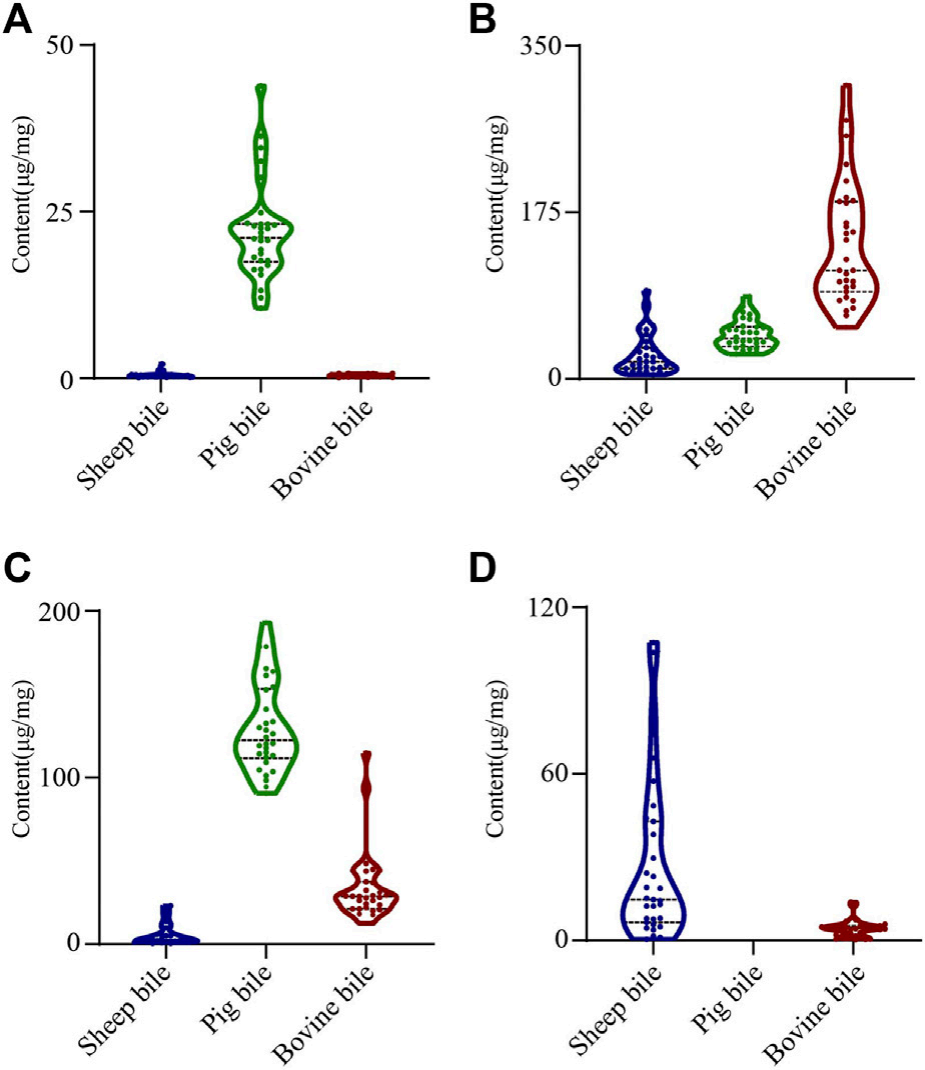
Violin plots showing the content of individual key feature marker in samples of bile from three animals. **(A)** TDCA; **(B)** GCA; **(C)** GCDCA; **(D)** CA.

To further elucidate the distribution of the four key feature markers in the bile of various animal species, UPLC-QQQ-MS was used to quantify the content of the four components. The concentration of each bile acid was calculated from its linear standard curve, which was plotted with the peak area as the vertical coordinate and the concentration of the standard solution as the horizontal coordinate. All standard curves showed good linearity, with coefficients of determination (R^2^) ranging from 0.9885 to 0.9978 (Table 1). Furthermore, violin plots of each key feature marker in the bile of the three animals were shown (Figure 3). A violin plot is usually used to show the state of the distribution in multiple datasets and the probability density. This plot combines the features of box plots and density plots, which can show the distribution of the data (Kurasiak-Popowska et al., 2020). The results showed that there were significant differences in the contents of four bile acids in the various types of animals. GCDCA and TDCA were highest in pig bile; GCA was highest in bovine bile; and CA was highest in sheep bile, which was consistent with previous studies (Chen et al., 2021). Therefore, these results could be taken into account when extracting bile acids from bile to make the use of bile more effective and targeted.

**TABLE 1:** Mass spectrum information of key feature markers by UPLC-QQQ-MS.

Compound	Retention time (min)	Qualifier and quantifier MRM transitions		DP (volts)	CE (volts)	R^2^
		Q1	Q3			
CA	5.55	407.30	343.40	−130	−46	0.9885
GCA	4.90	464.60	464.60	−140	−15	0.9980
TDCA	7.29	498.30	498.30	−150	−15	0.9978
GCDCA	5.61	448.60	448.60	−208	−15	0.9960

### 3.5 Anti-inflammatory activity of key feature components

Four key feature markers were validated by machine learning, clarifying the effectiveness of TDCA, GCA, CA, and GCDCA in differentiating the bile of the three animals. Therefore, anti-inflammatory activity assays were performed on these four bile acids to further evaluate their biological activity. It is hoped that the activity of the key markers will further reveal the differences in the pharmacological effects of the various bile. This could provide a strong basis for the selection of bile for later processing.

#### 3.5.1 Effect of each key feature marker on NF-κB activity in 293T cells

Cytokine expression, apoptosis regulation, and cell proliferation are highly dependent on the transcription factor NF-κB, which plays a key role in the regulation of the inflammatory response (Emam et al., 2021). In this study, the viability of 293T cells was assessed after treatment with different concentrations of four key feature markers (Supplementary Figure S2). GCDCA and TDCA did not show cytotoxic effects on 293T cells at the test concentrations of 10^−2^–10^2^ μM; GCA and CA concentrations of 10^−5^–10^–3^ μM and 10^−5^–10^–1^ μM, respectively, showed no cytotoxic effect on 293T cells. TNF-α was used as the modelling drug and 10 μg/mL of TNF-α complete medium solution was added to the model group; positive drug and different concentrations of bile acid solutions were prepared using 10 μg/mL of TNF-α complete medium solution and the prepared solutions of each group were added to the cells of the corresponding group. Then, the cells were treated with the maximum nontoxic concentrations of each key marker and evaluated for anti-inflammatory effects. The nuclear factor NF-κB pathway has long been recognized as a classic proinflammatory signaling pathway. This was mainly based on the role of NF-κB in the expression of proinflammatory genes, including cytokines, chemokines, and adhesion molecules (Lawrence 2009). Dual luciferase reporter gene assay systems were used to determine the effect of key feature markers on the expression levels of NF-κB in cells. As shown in Supplementary Figure S3, the positive control medicine dexamethasone (10 μM) significantly inhibited TNF-α-induced NF-κB production (*p* < 0.01), and TDCA, GCA, CA, and GCDCA also had a significant inhibitory effect on NF-κB. This finding suggested that four bile acids were potential NF-κB inhibitors, with preliminary confirmation of their anti-inflammatory activity.

#### 3.5.2 Effect of key markers on NO production in RAW264.7 macrophages

In this study, the effects of four markers at concentrations ranging from 10^−2^–10^2^ μM on the viability of RAW264.7 macrophage cells were first examined by the CCK-8 assay, and the results obtained are shown in Supplementary Figure S4. Compared with the control group, none of the four feature markers showed cytotoxicity to RAW264.7 cells at a concentration of 10^−2^–10^2^ μM. Therefore, 10^−2^–10^2^ μM of four markers was used to evaluate the anti-inflammatory effects.

**FIGURE 4:**
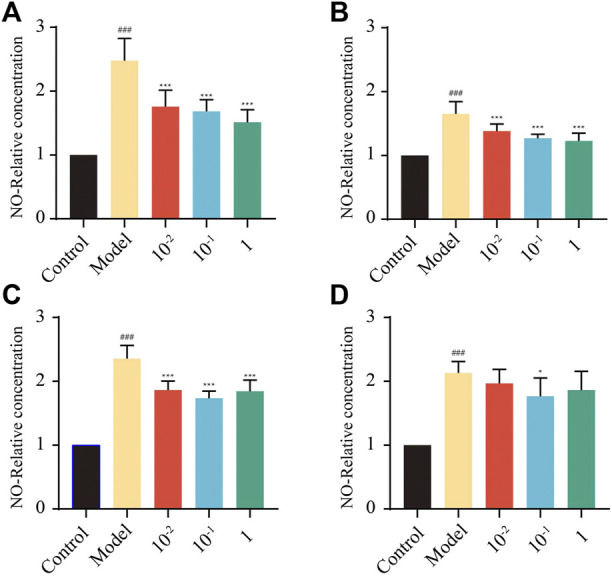
Expression of NO in RAW264.7 cells treated with different concentrations of key feature markers. **(A)** TDCA; **(B)** GCA; **(C)** GCDCA; **(D)** CA. (^##^ *p* < 0.01: Model vs. Control; *p < 0.05, **p < 0.01: Model vs. Treatment).

Macrophages can sense and respond to many stimuli and trigger inflammation by secreting NO. Excessive production of NO is associated with restriction of the immune response and remission of inflammation, and excessive NO production by macrophages has been observed in many inflammatory diseases (Lee et al., 2021). According to the results observed (Figures 4A–D), nontoxic doses of TDCA, GCA, and GCDCA significantly inhibited NO release compared to the LPS group (*p* < 0.01), as did 10^−1^ μM CA (*p* < 0.05). Four key feature markers could exert their anti-inflammatory activity in LPS-stimulated RAW264.7 cells by inhibiting NO production.

## 4 Conclusion

In this work, a strategy integrating metabolomics and machine learning was proposed for screening key feature markers that were closely associated with biliary anti-inflammatory activity. The metabolomics and machine learning approach were applied to identify four key feature markers. An SVM machine learning model was successfully developed to accurately evaluate unknown samples based on the content of each marker. Furthermore, cellular assays showed that all four key markers exhibited excellent anti-inflammatory activity against LPS-stimulated RAW264.7 cells. In brief, the screening of key feature markers related to anti-inflammatory function revealed the characteristics and pharmacological basis of various types of bile, thus providing a reasonable reference for exploring their pharmacological mechanisms.

## Data Availability

The original contributions presented in the study are included in the article/Supplementary Material, further inquiries can be directed to the corresponding authors.
