# Supplementary material for: An integrated strategy combining metabolomics and machine learning for the evaluation of bioactive markers that differentiate various bile
Source: Front Chem. 2022 Oct 19;10:1005843. doi: 10.3389/fchem.2022.1005843 (PMC9627196; doi:10.3389/fchem.2022.1005843)
Supplement: Supplementary file 1 [file DataSheet1.docx]

Supplementary material

# Supplementary Figures and Tables

## Supplementary Tables

**Supplementary Table 1** Chemical composition of animal bile in UPLC-Q-TOF/MS negative ESI mode

| No. | Rt(min) | Chemical formula | Theoretical m/z | Measured m/z | Compound | PubChem CID |
| --- | --- | --- | --- | --- | --- | --- |
| 1 | 4.13 | C_26_H_45_NO_7_S | 515.6951 | 514.2834 | Taurocholic acid | 6675 |
| 2 | 5.18 | C_26_H_45_NO_6_S | 499.6957 | 498.2885 | Taurodeoxycholic acid | 2733768 |
| 3 | 4.90 | C_26_H_43_NO_6_ | 465.6148 | 464.3011 | Glycocholic acid | 10140 |
| 4 | 6.67 | C_26_H_43_NO_5_ | 449.6154 | 448.3051 | Glycochenodeoxycholic acid | 22833540 |
| 5 | 7.13 | C_26_H_43_NO_5_ | 449.6154 | 448.3073 | Glycodeoxycholic acid | 3035026 |
| 6 | 6.38 | C_24_H_40_O_5_ | 408.5635 | 815.5669 | Cholic acid | 221493 |
| 7 | 4.04 | C_26_H_45_NO_6_S | 499.6957 | 498.2898 | Taurohyodeoxycholic acid | 119046 |
| 8 | 4.91 | C_26_H_45_NO_6_S | 499.6957 | 498.2892 | Taurochenodeoxycholic acid | 387316 |
| 9 | 4.91 | C_26_H_43_NO_5_ | 449.6154 | 448.3071 | Glycohyodeoxycholic acid | 114611 |
| 10 | 9.28 | C_24_H_40_O_4_ | 392.5641 | 783.5775 | Chenodeoxycholic acid | 10133 |

**Supplementary Table 2** Results of retention times of 10 molecular ion peaks in instrumental precision test investigations

|  | m/z | QC-1-1 | QC-1-2 | QC-1-3 | QC-1-4 | QC-1-5 | QC-1-6 | Average | RSD% |
| --- | --- | --- | --- | --- | --- | --- | --- | --- | --- |
| Peak 1 | 514.2839 | 4.06 | 4.04 | 4.04 | 4.04 | 4.04 | 4.04 | 4.04 | 0.20 |
| Peak 2 | 498.2887 | 5.24 | 5.23 | 5.23 | 5.22 | 5.22 | 5.20 | 5.22 | 0.26 |
| Peak 3 | 540.3302 | 13.51 | 13.48 | 13.49 | 13.49 | 13.49 | 13.48 | 13.49 | 0.08 |
| Peak 4 | 339.2325 | 16.68 | 16.68 | 16.68 | 16.68 | 16.68 | 16.68 | 16.68 | 0.00 |
| Peak 5 | 815.5677 | 6.38 | 6.38 | 6.38 | 6.38 | 6.38 | 6.38 | 6.38 | 0.00 |
| Peak 6 | 897.6192 | 6.68 | 6.65 | 6.68 | 6.68 | 6.66 | 6.67 | 6.67 | 0.19 |
| Peak 7 | 432.3113 | 10.19 | 10.18 | 10.17 | 10.18 | 10.19 | 10.18 | 17.68 | 0.04 |
| Peak 8 | 564.3302 | 12.26 | 12.24 | 12.23 | 12.23 | 12.21 | 12.23 | 12.23 | 0.13 |
| Peak 9 | 540.3302 | 13.51 | 13.48 | 13.49 | 13.49 | 13.49 | 13.48 | 18.68 | 0.06 |
| Peak 10 | 566.3458 | 14.15 | 14.14 | 14.14 | 14.14 | 14.14 | 14.14 | 14.14 | 0.03 |

**Supplementary Table 3** Results of the peak areas of 10 molecular ion peaks in the instrumental precision test examination

|  | m/z | QC-1-1 | QC-1-2 | QC-1-3 | QC-1-4 | QC-1-5 | QC-1-6 | Average | RSD% |
| --- | --- | --- | --- | --- | --- | --- | --- | --- | --- |
| Peak 1 | 514.2839 | 2707.20 | 2613.90 | 2708.75 | 2491.26 | 2687.39 | 2519.94 | 2621.41 | 3.68 |
| Peak 2 | 498.2887 | 1324.80 | 1459.63 | 1292.59 | 1314.53 | 1401.84 | 1233.88 | 1337.88 | 6.03 |
| Peak 3 | 540.3302 | 509.76 | 489.19 | 545.96 | 531.33 | 512.17 | 531.35 | 519.96 | 3.89 |
| Peak 4 | 339.2325 | 1940.97 | 1981.08 | 2186.48 | 2147.41 | 2157.90 | 2339.03 | 2125.48 | 6.84 |
| Peak 5 | 815.5677 | 119.52 | 105.96 | 111.86 | 113.19 | 111.79 | 108.53 | 111.81 | 4.12 |
| Peak 6 | 897.6192 | 220.73 | 241.90 | 231.18 | 246.78 | 254.94 | 241.28 | 239.47 | 5.02 |
| Peak 7 | 432.3113 | 300.96 | 327.51 | 347.81 | 312.77 | 323.68 | 290.80 | 317.25 | 6.41 |
| Peak 8 | 564.3302 | 278.74 | 263.42 | 306.16 | 274.31 | 302.80 | 271.85 | 282.88 | 6.18 |
| Peak 9 | 540.3302 | 509.76 | 489.19 | 545.96 | 531.33 | 512.17 | 531.35 | 519.96 | 3.89 |
| Peak 10 | 566.3458 | 365.44 | 366.68 | 366.59 | 355.87 | 351.34 | 325.66 | 355.26 | 4.46 |

**Supplementary Table 4** Results of the retention times of 10 molecular ion peaks in a method reproducibility test investigation

|  | m/z | QC-1 | QC-2 | QC-3 | QC-4 | QC-5 | QC-6 | Average | RSD% |
| --- | --- | --- | --- | --- | --- | --- | --- | --- | --- |
| Peak 1 | 514.2839 | 4.06 | 4.04 | 4.06 | 4.06 | 4.06 | 4.04 | 4.05 | 0.25 |
| Peak 2 | 512.2675 | 3.74 | 3.73 | 3.73 | 3.73 | 3.74 | 3.73 | 3.73 | 0.14 |
| Peak 3 | 453.2847 | 6.38 | 6.38 | 6.38 | 6.38 | 6.38 | 6.38 | 0.00 | 0.00 |
| Peak 4 | 498.2887 | 5.24 | 5.20 | 5.22 | 5.22 | 5.22 | 5.22 | 5.22 | 0.24 |
| Peak 5 | 897.6192 | 6.68 | 6.67 | 6.66 | 6.67 | 6.67 | 6.67 | 6.67 | 0.10 |
| Peak 6 | 432.3113 | 10.19 | 10.17 | 10.18 | 10.17 | 10.18 | 10.17 | 10.18 | 0.08 |
| Peak 7 | 564.3302 | 12.15 | 12.24 | 12.23 | 12.22 | 12.22 | 12.23 | 12.22 | 0.27 |
| Peak 8 | 540.3302 | 13.51 | 13.49 | 13.47 | 13.48 | 13.48 | 13.48 | 13.49 | 0.10 |
| Peak 9 | 566.3458 | 14.15 | 14.15 | 14.14 | 14.14 | 14.15 | 14.14 | 14.15 | 0.04 |
| Peak 10 | 568.3612 | 15.67 | 15.67 | 15.67 | 15.66 | 15.67 | 15.66 | 15.67 | 0.03 |

**Supplementary Table 5** Results of peak areas of 10 molecular ion peaks in a method reproducibility test examination

|  | m/z | QC-1 | QC-2 | QC-3 | QC-4 | QC-5 | QC-6 | Average | RSD% |
| --- | --- | --- | --- | --- | --- | --- | --- | --- | --- |
| Peak 1 | 514.2839 | 2707.20 | 2430.37 | 2953.95 | 2764.96 | 2738.50 | 2472.09 | 2677.85 | 7.32 |
| Peak 2 | 512.2675 | 132.22 | 136.38 | 148.11 | 123.61 | 144.86 | 112.08 | 132.88 | 10.13 |
| Peak 3 | 453.2847 | 151.58 | 144.34 | 142.28 | 131.27 | 144.72 | 123.79 | 139.66 | 7.29 |
| Peak 4 | 498.2887 | 1324.80 | 1332.16 | 1269.35 | 1348.08 | 1155.85 | 1211.61 | 1273.64 | 6.01 |
| Peak 5 | 897.6192 | 220.73 | 246.49 | 230.70 | 238.82 | 248.68 | 198.90 | 230.72 | 8.11 |
| Peak 6 | 432.3113 | 300.96 | 325.70 | 303.34 | 288.24 | 316.76 | 303.81 | 306.47 | 4.27 |
| Peak 7 | 564.3302 | 278.74 | 259.23 | 279.01 | 275.44 | 295.15 | 309.31 | 282.81 | 6.12 |
| Peak 8 | 540.3302 | 509.76 | 563.65 | 536.38 | 579.19 | 555.55 | 518.50 | 543.84 | 4.96 |
| Peak 9 | 566.3458 | 365.44 | 317.81 | 396.51 | 387.65 | 352.58 | 343.07 | 360.51 | 8.08 |
| Peak 10 | 568.3612 | 335.39 | 318.12 | 393.58 | 359.40 | 425.67 | 361.99 | 365.69 | 10.67 |

**Supplementary Table 6** Results of the retention times of 10 molecular ion peaks in a sample stability test investigation

|  | m/z | QC-0 h | QC-6 h | QC-12 h | QC-18 h | Average | RSD% |
| --- | --- | --- | --- | --- | --- | --- | --- |
| Peak 1 | 514.2839 | 4.06 | 4.04 | 4.04 | 4.04 | 4.05 | 0.25 |
| Peak 2 | 512.2675 | 3.74 | 3.73 | 3.74 | 3.74 | 3.74 | 0.13 |
| Peak 3 | 498.2887 | 4.03 | 4.01 | 4.01 | 4.00 | 4.01 | 0.31 |
| Peak 4 | 498.2887 | 5.00 | 4.97 | 4.98 | 4.96 | 4.98 | 0.34 |
| Peak 5 | 453.2847 | 5.77 | 5.74 | 5.75 | 5.75 | 5.75 | 0.22 |
| Peak 6 | 897.6192 | 6.69 | 6.68 | 6.65 | 6.68 | 6.68 | 0.26 |
| Peak 7 | 432.3113 | 10.19 | 10.16 | 10.17 | 10.18 | 10.18 | 0.13 |
| Peak 8 | 564.3302 | 12.24 | 12.22 | 12.22 | 12.23 | 12.23 | 0.08 |
| Peak 9 | 566.3458 | 14.16 | 14.15 | 14.13 | 14.15 | 14.15 | 0.09 |
| Peak 10 | 540.3302 | 13.49 | 13.48 | 13.47 | 13.47 | 13.48 | 0.07 |

**Supplementary Table 7** Results of the peak areas of 10 molecular ion peaks in the sample stability test investigation

|  | m/z | QC-0 h | QC-6 h | QC-12 h | QC-18 h | Average | RSD% |
| --- | --- | --- | --- | --- | --- | --- | --- |
| Peak 1 | 514.2839 | 2707.20 | 2776.85 | 2553.38 | 2460.74 | 2624.54 | 5.47 |
| Peak 2 | 512.2675 | 132.22 | 142.53 | 142.71 | 151.20 | 142.17 | 5.46 |
| Peak 3 | 498.2887 | 412.64 | 399.26 | 416.12 | 446.64 | 418.66 | 4.78 |
| Peak 4 | 498.2887 | 477.29 | 380.62 | 506.27 | 500.69 | 466.21 | 12.53 |
| Peak 5 | 453.2847 | 151.58 | 139.12 | 143.00 | 143.73 | 144.36 | 3.62 |
| Peak 6 | 897.6192 | 220.73 | 223.69 | 239.76 | 239.23 | 230.85 | 4.36 |
| Peak 7 | 432.3113 | 300.96 | 315.24 | 323.40 | 366.29 | 326.47 | 8.61 |
| Peak 8 | 564.3302 | 278.74 | 312.83 | 310.26 | 307.91 | 302.44 | 5.26 |
| Peak 9 | 566.3458 | 365.44 | 367.58 | 381.01 | 359.54 | 368.39 | 2.46 |
| Peak 10 | 540.3302 | 509.76 | 535.88 | 528.56 | 595.45 | 542.41 | 6.83 |

**Supplementary Table 8** Results of the SVM machine learning model

| Polynomial coefficients | coef0 | CV accuracy (%) | Classified accuracy (%) |
| --- | --- | --- | --- |
| 2 | 1.35 | 96.70 | 100.00 |
| 3 | 7.40 | 100.00 | 100.00 |
| 4 | 5.40 | 100.00 | 100.00 |

## Supplementary Figures


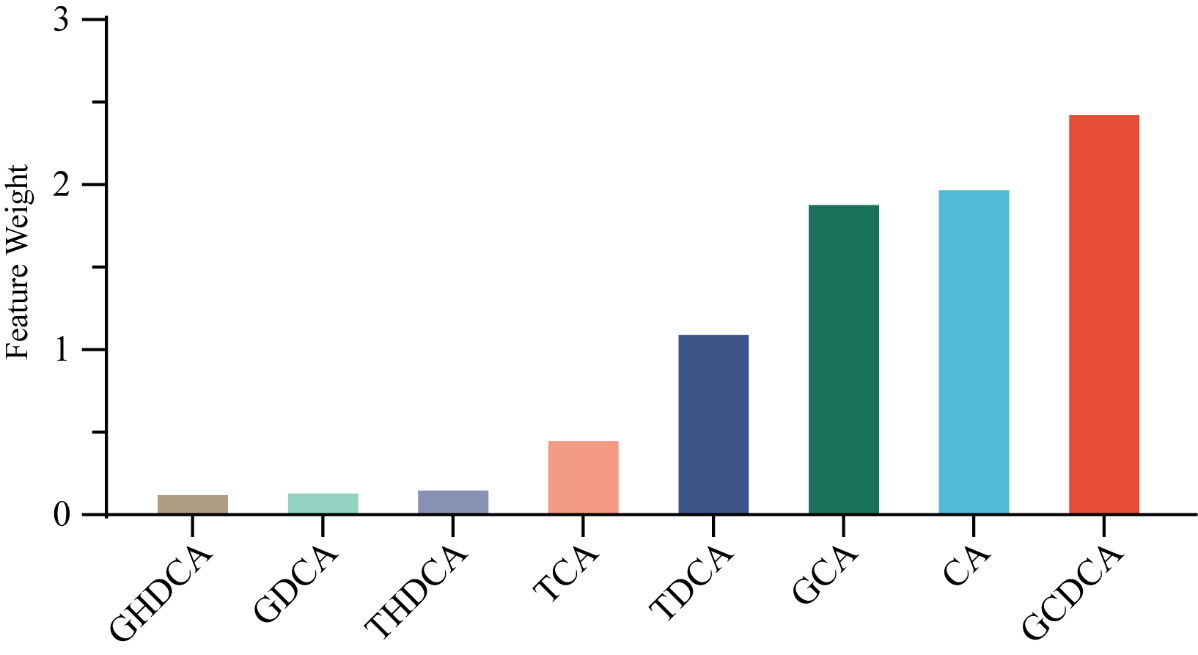


**Supplementary Figure 1** Results of NCA


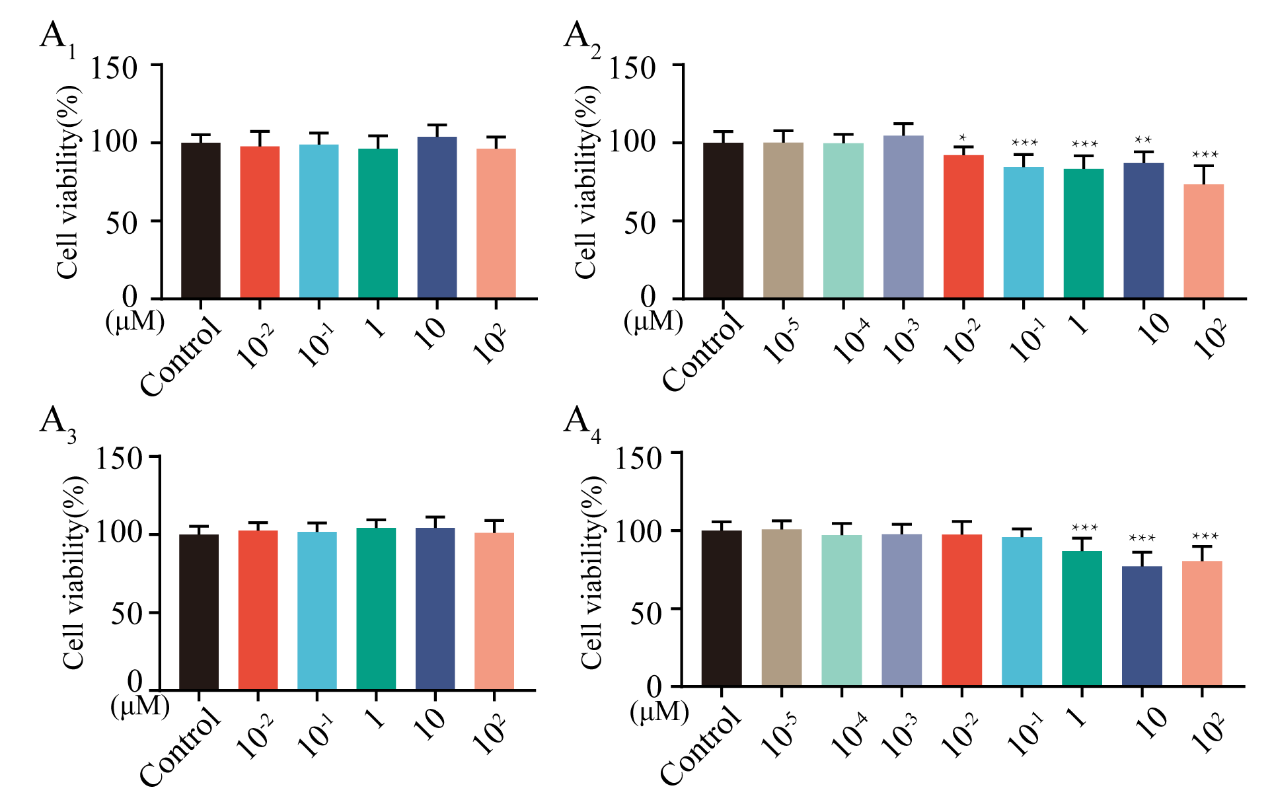


**Supplementary Figure 2** Activity of 293T cells treated with different concentrations of the key feature markers. A_1_ TDCA; A_2_ GCA; A_3_ GCDCA; A_4_ CA.(**p*<0.05, ***p*<0.01, ****p*<0.001:Control vs Treatment)


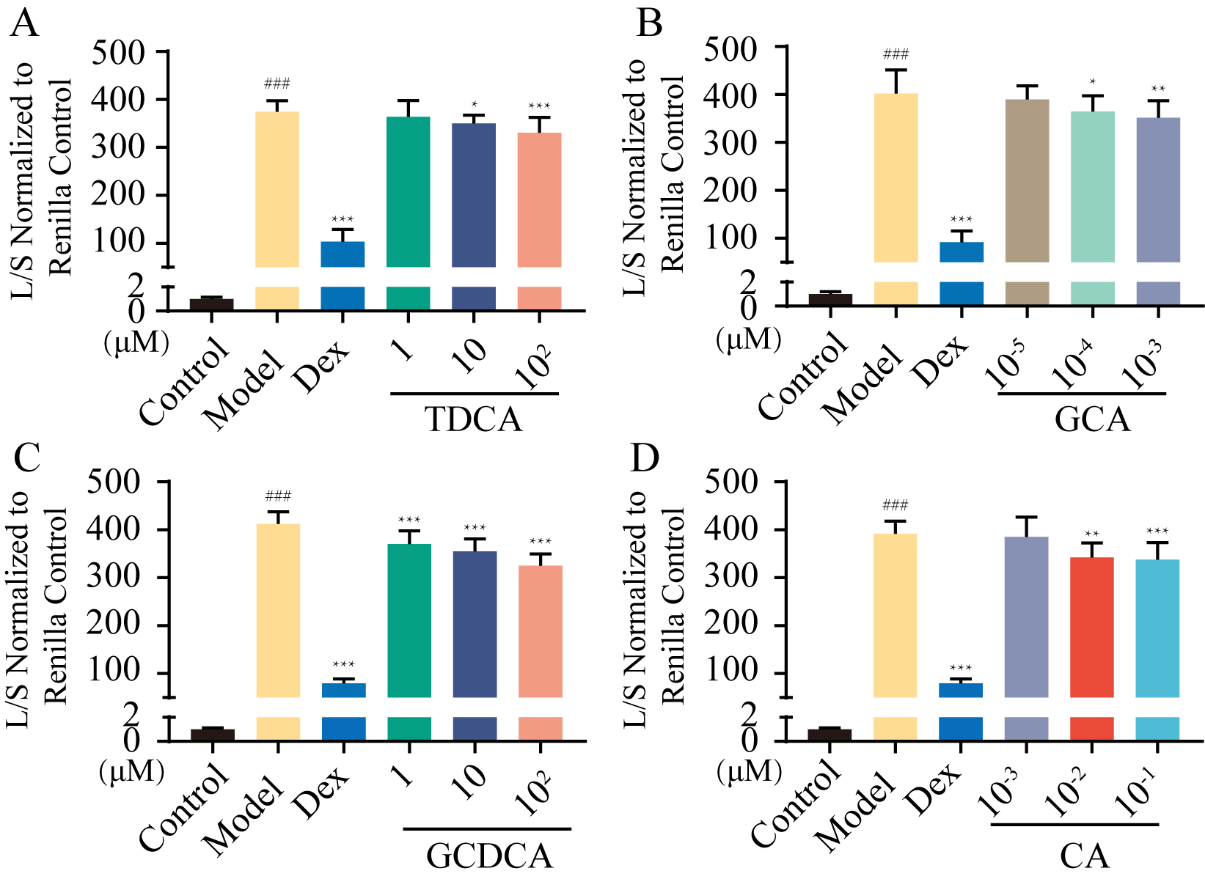


**Supplementary Figure 3** A-D Confirmation of the biological activity of key feature components by a dual luciferase reporter assay system. (###p<0.0001: Model vs Control ; **p*<0.05, ***p*<0.01, ****p*<0.001: Model vs Treatment)


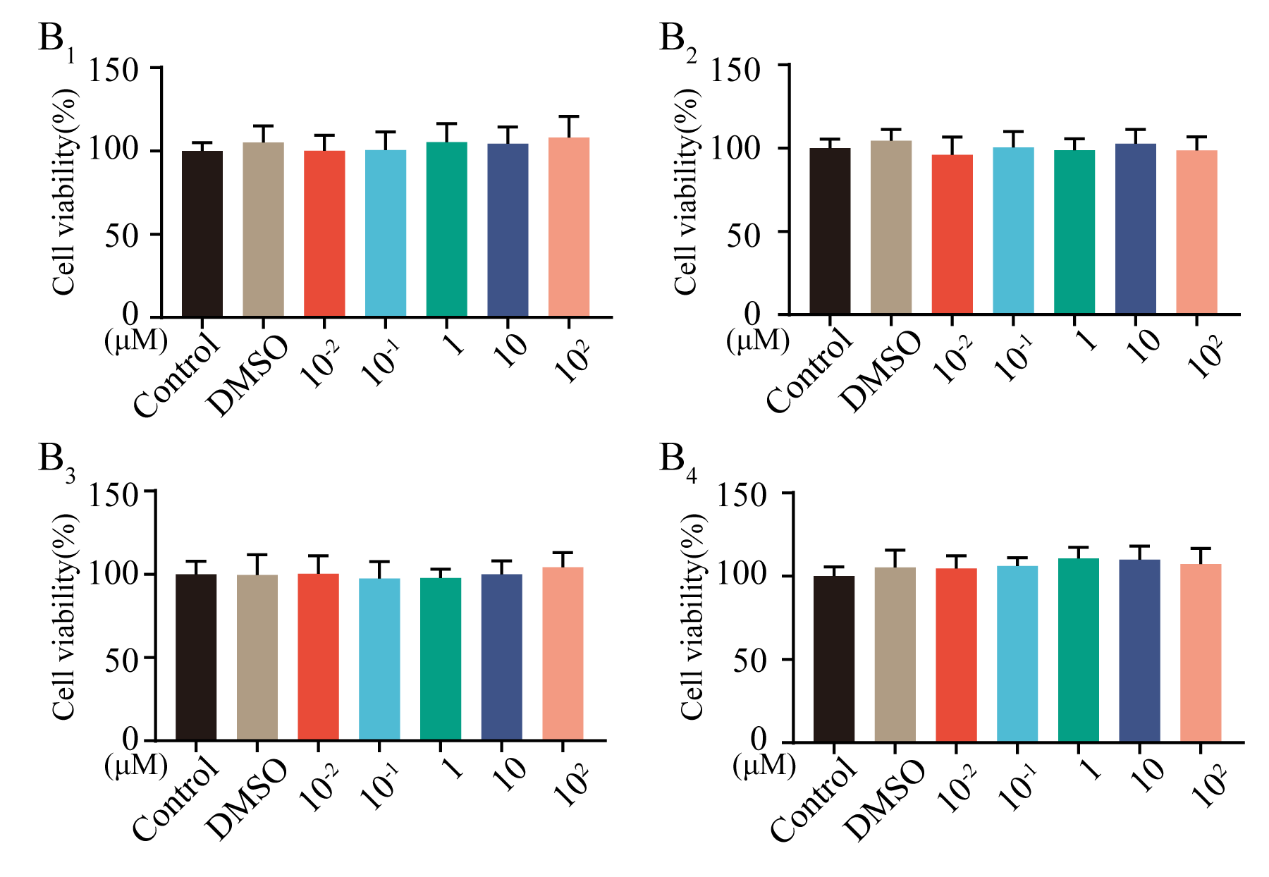


**Supplementary Figure 4** Activity of RAW264.7 cells treated with different concentrations of the key feature markers. B_1_ TDCA; B_2_ GCA; B_3_ GCDCA; B_4_ CA.
